# Overview and Evaluation of Bluetooth Low Energy: An Emerging Low-Power Wireless Technology

**DOI:** 10.3390/s120911734

**Published:** 2012-08-29

**Authors:** Carles Gomez, Joaquim Oller, Josep Paradells

**Affiliations:** 1 Universitat Politècnica de Catalunya/Fundació i2Cat, C/Esteve Terradas, 7, Castelldefels 08860, Spain; 2 Universitat Politècnica de Catalunya/Fundació i2Cat, C/Jordi Girona, 1-3, Barcelona 08034, Spain; E-Mails: joaquim.oller@entel.upc.edu (J.O.); josep.paradells@entel.upc.edu (J.P.)

**Keywords:** Bluetooth Low Energy, sensor networks, Internet of Things

## Abstract

Bluetooth Low Energy (BLE) is an emerging low-power wireless technology developed for short-range control and monitoring applications that is expected to be incorporated into billions of devices in the next few years. This paper describes the main features of BLE, explores its potential applications, and investigates the impact of various critical parameters on its performance. BLE represents a trade-off between energy consumption, latency, piconet size, and throughput that mainly depends on parameters such as *connInterval* and *connSlaveLatency*. According to theoretical results, the lifetime of a BLE device powered by a coin cell battery ranges between 2.0 days and 14.1 years. The number of simultaneous slaves per master ranges between 2 and 5,917. The minimum latency for a master to obtain a sensor reading is 676 μs, although simulation results show that, under high bit error rate, average latency increases by up to three orders of magnitude. The paper provides experimental results that complement the theoretical and simulation findings, and indicates implementation constraints that may reduce BLE performance.

## 1. Introduction

Bluetooth Low Energy (BLE) is an emerging wireless technology developed by the Bluetooth Special Interest Group (SIG) for short-range communication. In contrast with previous Bluetooth flavors, BLE has been designed as a low-power solution for control and monitoring applications. BLE is the distinctive feature of the Bluetooth 4.0 specification [1].

The advent of BLE has occurred while other low-power wireless solutions, such as ZigBee, 6LoWPAN or Z-Wave, have been steadily gaining momentum in application domains that require multihop networking [2,3]. However, BLE constitutes a single-hop solution applicable to a different space of use cases in areas such as healthcare, consumer electronics, smart energy and security.

The widespread use of Bluetooth technology (e.g., in mobile phones, laptops, automobiles, *etc.*) may fuel adoption of BLE, since implementation of the latter can leverage similarities with classic Bluetooth. According to published forecasts [4], BLE is expected to be used in billions of devices in the near future. In fact, the IETF 6LoWPAN Working Group (WG) [5] has already recognized the importance of BLE for the Internet of Things. As of the writing of this article, the 6LoWPAN WG is developing a specification for the transmission of IPv6 packets over BLE [6].

This paper describes the main features of BLE, investigates the impact of critical parameters on its performance, and explores its potential applications. The rest of the paper is organized as follows: Section 2 overviews the BLE protocol stack and describes the operation and main characteristics of each layer; Section 3 evaluates the energy consumption, latency and network size of BLE and discusses application layer BLE throughput; Section 4 explores the application and market adoption possibilities for BLE, and provides a comparison with other wireless low-power technologies. Finally Section 5 concludes the paper with the main remarks.

## 2. Bluetooth Low Energy Protocol Stack

This section presents the BLE protocol stack, and describes the main mechanisms and features of each layer.

### 2.1. BLE Protocol Stack Overview

**Figure 1.**
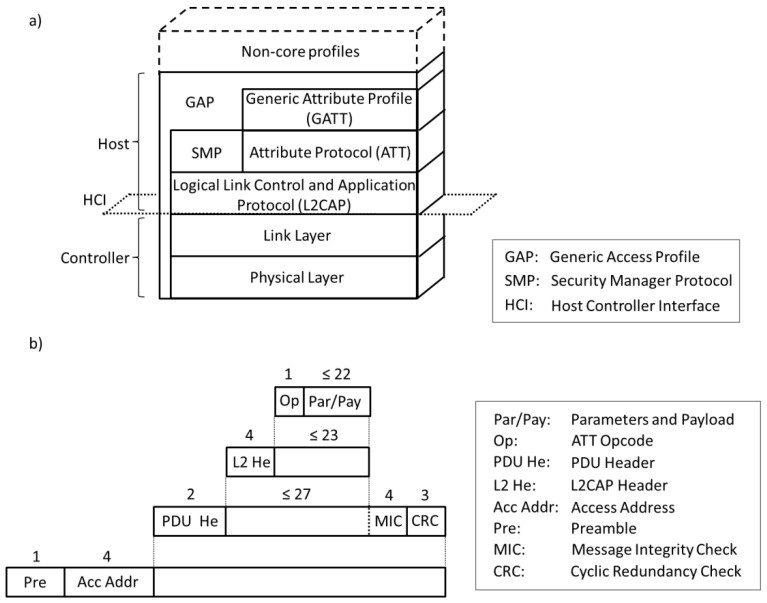
(**a**) BLE protocol stack; (**b**) structure of a BLE data unit (note: the size of each field is expressed in bytes).

Like in classic Bluetooth [7], the BLE protocol stack is composed of two main parts: the Controller and the Host. The Controller comprises the Physical Layer and the Link Layer, and is typically implemented as a small System-on-Chip (SOC) with an integrated radio. The Host runs on an application processor and includes upper layer functionality, *i.e.*, the Logical Link Control and Adaptation Protocol (L2CAP), the Attribute Protocol (ATT), the Generic Attribute Profile (GATT), the Security Manager Protocol (SMP) and the Generic Access Profile (GAP). Communication between the Host and the Controller is standardized as the Host Controller Interface (HCI). Finally, non-core profiles (*i.e.*, application layer functionality not defined by the Bluetooth specification) can be used on top of the Host. Figure 1(a) illustrates the BLE protocol stack. Figure 1(b) depicts the structure and size of the different fields contributed by each layer to a Physical Layer data unit when application data are transmitted. Subsections 2.2 to 2.8 focus on each layer of the BLE protocol stack.

Although some of the BLE Controller features are inherited from the classic Bluetooth Controller, both types of Controller are currently incompatible. Hence, a device that only implements BLE (which is referred to as a *single-mode* device) cannot communicate with a device that only implements classic Bluetooth. It is expected that many devices will implement both the classic Bluetooth and the BLE protocol stacks. These devices are called *dual-mode* devices.

### 2.2. Physical Layer

BLE operates in the 2.4 GHz Industrial Scientific Medical (ISM) band and defines 40 Radio Frequency (RF) channels with 2 MHz channel spacing. There are two types of BLE RF channels: advertising channels and data channels. Advertising channels are used for device discovery, connection establishment and broadcast transmission, whereas data channels are used for bidirectional communication between connected devices.

Three channels are defined as advertising channels. These channels have been assigned center frequencies that minimize overlapping with IEEE 802.11 channels 1, 6 and 11, which are commonly used in several countries.

An adaptive frequency hopping mechanism is used on top of the data channels in order to face interference and wireless propagation issues, such as fading and multipath. This mechanism selects one of the 37 available data channels for communication during a given time interval.

All physical channels use a Gaussian Frequency Shift Keying (GFSK) modulation, which is simple to implement. The modulation index is in the range between 0.45 and 0.55, which allows reduced peak power consumption. The physical layer data rate is 1 Mbps.

The receiver sensitivity is defined in BLE as the signal level at the receiver for which a Bit Error Rate (BER) of 10^−3^ is achieved. The BLE specification mandates a sensitivity better than or equal to −70 dBm. The coverage range is typically over various tens of meters.

### 2.3. Link Layer

In BLE, when a device only needs to broadcast data, it transmits the data in advertising packets through the advertising channels. Any device that transmits advertising packets is called an advertiser. The transmission of packets through the advertising channels takes place in intervals of time called advertising events. Within an advertising event, the advertiser sequentially uses each advertising channel for packet transmission. Devices that only aim at receiving data through the advertising channels are called scanners.

Bidirectional data communication between two devices requires them to connect to each other. The creation of a connection between two devices is an asymmetric procedure by which an advertiser announces through the advertising channels that it is a connectable device, while the other device (referred to as an initiator) listens for such advertisements. When an initiator finds an advertiser, it may transmit a Connection Request message to the advertiser, which creates a point-to-point connection between the two devices. Both devices can then communicate by using the physical data channels. The packets for this connection will be identified by a randomly generated 32-bit access code.

BLE defines two device roles at the Link Layer for a created connection: the master and the slave. These are the devices that act as initiator and advertiser during the connection creation, respectively. A master can manage multiple simultaneous connections with different slaves, whereas each slave can only be connected to one master. Thus, the network composed by a master and its slaves, which is called a piconet, follows a star topology. Currently, a BLE device can only belong to one piconet.

In order to save energy, slaves are in sleep mode by default and wake up periodically to listen for possible packet receptions from the master. The master determines the instants in which slaves are required to listen, and thus coordinates the medium access by using a Time Division Multiple Access (TDMA) scheme. The master also provides the slave with the information needed for the frequency hopping algorithm (including the map of data channels to be used) and for the connection supervision. The parameters related with the management of a connection are transmitted in the Connection Request message and can be updated during the connection for various reasons (e.g., using a new data channel map due to a change of the interference pattern).

Once a connection between a master and a slave is created, the physical channel is divided into non-overlapping time units called connection events. Within a connection event, all packets are transmitted using the same data channel frequency. Every connection event starts with the transmission of a packet by the master. If the slave receives a packet, the slave must send a packet to the master in response. However, the master is not required to send a packet upon receipt of a packet from the slave. At least, an Inter Frame Space (IFS) of 150 μs must pass between the end of the transmission of a packet and the start of the next one. While master and slave continue to alternate in sending packets, the connection event is considered to be open. Data channel packets include a More Data (MD) bit which signals whether the sender has more information to transmit. If none of the devices has more data to transmit, the connection event will be closed and the slave will not be required to listen until the beginning of the next connection event. Other circumstances that force the end of a connection event include the reception of two consecutive packets with bit errors by either the master or the slave, and the corruption of the access address field of a packet sent by any device. In order to allow bit error detection, all data units include a 24-bit Cyclic Redundancy Check (CRC) code.

For a new connection event, master and slave use a new data channel frequency, which is computed by using the frequency hopping algorithm. The time between the start of two consecutive connection events is specified by the *connInterval* parameter, which is a multiple of 1.25 ms in the range between 7.5 ms and 4 s. Another important parameter is *connSlaveLatency*, which defines the number of consecutive connection events during which the slave is not required to listen to the master and thus can keep the radio turned off. This parameter is an integer between 0 and 499 and should not cause a supervision timeout. A supervision timeout happens when the time since the last received packet exceeds the *connSupervisionTimeout* parameter, which is in the range between 100 ms and 32 s. The purpose of this mechanism is to detect the loss of a connection due to severe interference or the movement of a device outside the range of its peer.

Link Layer connections use a stop-and-wait flow control mechanism based on cumulative acknowledgments, which at the same time provides error recovery capabilities. Each data channel packet header contains two one-bit fields called the Sequence Number (SN) and the Next Expected Sequence Number (NESN). The SN bit identifies the packet, whereas the NESN indicates which packet from the peer device should be received next. If a device successfully receives a data channel packet, the NESN of its next packet will be incremented, and that packet will serve as an acknowledgement. Otherwise, if a device receives a packet with an invalid CRC check, the NESN of the received packet cannot be relied upon. This forces the receiving device to resend its last transmitted packet, which serves as a negative acknowledgement.

### 2.4. L2CAP

The L2CAP used in BLE is an optimized and simplified protocol based on the classic Bluetooth L2CAP. In BLE, the main goal of L2CAP is to multiplex the data of three higher layer protocols, ATT, SMP and Link Layer control signaling, on top of a Link Layer connection. The data of these services are handled by L2CAP in a best-effort approach and without the use of retransmission and flow control mechanisms, which are available in other Bluetooth versions. Segmentation and reassembly capabilities are not used, since upper layer protocols provide data units that fit into the maximum L2CAP payload size, which is equal to 23 bytes in BLE.

### 2.5. ATT

The ATT defines the communication between two devices playing the roles of server and client, respectively, on top of a dedicated L2CAP channel. The server maintains a set of attributes. An attribute is a data structure that stores the information managed by the GATT, the protocol that operates on top of the ATT. The client or server role is determined by the GATT, and is independent of the slave or master role.

The client can access the server's attributes by sending requests, which trigger response messages from the server. For greater efficiency, a server can also send to a client two types of unsolicited messages that contain attributes: (i) notifications, which are unconfirmed; and (ii) indications, which require the client to send a confirmation. A client may also send commands to the server in order to write attribute values. Request/response and indication/confirmation transactions follow a stop-and-wait scheme.

### 2.6. GATT

The GATT defines a framework that uses the ATT for the discovery of services, and the exchange of characteristics from one device to another. A characteristic is a set of data which includes a value and properties. The data related to services and characteristics are stored in attributes. For example, a server that runs a ‘temperature sensor’ service may account with a ‘temperature’ characteristic that uses an attribute for describing the sensor, another attribute for storing temperature measurement values and a further attribute for specifying the measurement units.

### 2.7. Security

BLE offers various security services for protecting the information exchange between two connected devices. Most of the supported security services can be expressed in terms of two mutually-exclusive security modes called *LE Security Mode 1* and *LE Security Mode 2*. These two modes provide security functionality at the Link Layer and at the ATT layer, respectively.

The BLE Link Layer supports encryption and authentication by using the Cipher Block Chaining-Message Authentication Code (CCM) algorithm [8] and a 128-bit AES block cipher. When encryption and authentication are used in a connection, a 4-byte Message Integrity Check (MIC) is appended to the payload of the data channel PDU (see Figure 1(b)). Encryption is then applied to the PDU payload and MIC fields.

It is also possible to transmit authenticated data over an unencrypted Link Layer connection. In this case, a 12-byte signature is placed after the data payload at the ATT layer. The signature is computed by applying an algorithm that uses 128-bit AES as the block cipher [1]. One input to the algorithm is a counter, which is used in order to provide protection against replay attacks. If the receiver verifies the signature, it assumes that the data have been sent by the trusted source.

In addition to the described services, BLE supports a mechanism called privacy feature, which allows a device to use private addresses and frequently change them. The privacy feature mitigates the threat by which an adversary can track a BLE device. The private addresses are generated by encrypting the public address of the device, which can be resolved by a trusted device that has been provided with the corresponding encryption key.

Each security mode accounts with different levels, which express requirements as to the type of *pairing* that has to be used. Pairing is a procedure by which the devices generate and distribute key material. Table 1 summarizes the security services and the type of pairing (if any) required by each security mode and level.

Pairing comprises three phases. In the first phase, the two connected devices announce their input/output capabilities and, based on these, they choose a suitable method for the second phase.

The second phase has the purpose of generating the Short-Term Key (STK), which will be used in the third phase to secure the distribution of key material. In the second phase, the pairing devices first agree on a Temporary Key (TK), by means of the Out Of Band, the Passkey Entry or the Just Works methods. The Out of Band method uses out of band communication means (e.g., NFC [9]) for the TK agreement. In the Passkey Entry method, the user passes six numeric digits as the TK between the devices. When none of the first two methods can be used, the Just Works method is employed, although it is not authenticated and it does not provide protection against Man In The Middle (MITM) attacks [10]. Based on the TK, and on random values generated by each pairing device, the STK is obtained by both devices, which leads to the end of the second phase.

In the third phase, each endpoint of the connection may distribute to the other endpoint up to three 128-bit keys called the Long-Term Key (LTK), the Connection Signature Resolving Key (CSRK) and the Identity Resolving Key (IRK). The LTK is used to generate the 128-bit key employed for Link Layer encryption and authentication. The CSRK is used for the data signing performed at the ATT layer. The third key (*i.e.*, the IRK), is used to generate a private address on the basis of a device public address. The message exchange required for distributing the LTK, the CSRK or the IRK is encrypted by using the STK obtained in the second phase.

The Security Manager Protocol (SMP) carries out the message exchange of the three described pairing phases. SMP operates on top of a fixed L2CAP channel.

A vulnerability that currently exists in BLE is the fact that none of the pairing methods is protected against passive eavesdropping. Therefore, an adversary who obtains the pairing messages can determine the LTK, the CSRK or the IRK. However, according to the Bluetooth 4.0 specification, a future BLE version will use elliptic curve cryptography and Diffie-Hellman public key exchanges in order to solve the described vulnerability issue [1].

### 2.8. GAP and Application Profiles

At the highest level of the core BLE stack, the GAP specifies device roles, modes and procedures for the discovery of devices and services, the management of connection establishment and security.

The BLE GAP defines four roles with specific requirements on the underlying controller: Broadcaster, Observer, Peripheral and Central. A device in the Broadcaster role only broadcasts data (via the advertising channels) and does not support connections with other devices. The Observer role is complementary for the Broadcaster, *i.e.*, it has the purpose of receiving the data transmitted by the Broadcaster. The Central role is designed for a device that is in charge of initiating and managing multiple connections, whereas the Peripheral role is designed for simple devices which use a single connection with a device in the Central role. In consequence, the Central and Peripheral roles require that the device's controller support the master and slave roles, respectively. A device may support various roles, but only one role can be adopted at a given time.

Finally, since certain types of applications may benefit from reusing common functionality, additional profiles can be built on top of the GAP. Bluetooth follows a profile hierarchy, whereby a new profile including all the requirements of an existing profile can be defined. A highest-level profile that specifies how applications can interoperate is called an application profile. Application profiles, which are also specified by the Bluetooth SIG, favor interoperability between devices from different manufacturers.

## 3. Performance Evaluation

This section evaluates the performance of BLE in terms of energy consumption, latency and piconet size, for various use cases and configurations, and discusses application layer BLE throughput. The size of the notification, command and response messages considered in this study is the maximum one (*i.e.*, 37 bytes at the Physical Layer), whereas polls sent by the master and acknowledgments are assumed to be empty PDUs (*i.e.*, PDUs without payload). Latency results have been obtained by exploiting simulation tools that we developed for this purpose (given that current simulators do not support BLE yet), and have been complemented by means of experimental measurements. The simulation tools model two connected BLE devices that communicate with each other, taking into account all the BLE stack layers and their behavior in the presence of bit errors. Piconet size results have been obtained theoretically, whereas energy consumption has been analyzed both theoretically and empirically. Further details about the evaluation methods are provided in each corresponding subsection.

### 3.1. Energy Consumption

We first investigate the theoretical lifetime of a slave that is connected to a master in a data collection application. Note that a slave is typically a device with limited energy supply, whereas a master may not suffer the same energy constraints. We consider two different methods by which the master obtains sensor measurement readings (which are handled as attribute values) from the slave, which is assumed to act as the attribute server. We denote these methods by one-way ATT communication and round-trip ATT dialogue, respectively. In the one-way ATT communication, the slave sends a notification in response to a poll from the master. In the round-trip ATT dialogue, the master sends a request to the slave, which transmits a response to the master (and both the request and the response trigger Link Layer acknowledgments). For the two methods described, the first packet transmission from the master takes place at the beginning of each connection event.

The evaluation is carried out theoretically by assuming current consumption values obtained from measurements for the CC2540 radio chip, for a transmit power of 0 dBm [11]. Specifically, for the one-way ATT communication, the study takes into account the energy consumed during each one of the following states: device wake up, radio turn on (in order to receive the initial BLE packet from the master), request reception, radio switch to transmit mode, notification transmission, and final post-processing before the device returns to sleep mode. For the round-trip ATT dialogue, the additional energy consumption due to response transmission, radio switch to receive mode, and acknowledgment reception is also considered. The energy consumption during sleep periods is considered as well for both types of ATT transactions. For the evaluation of the device lifetime, we assume an ideal battery with a capacity of 230 mAh (*i.e.*, a common value for a coin cell battery).

**Figure 2.**
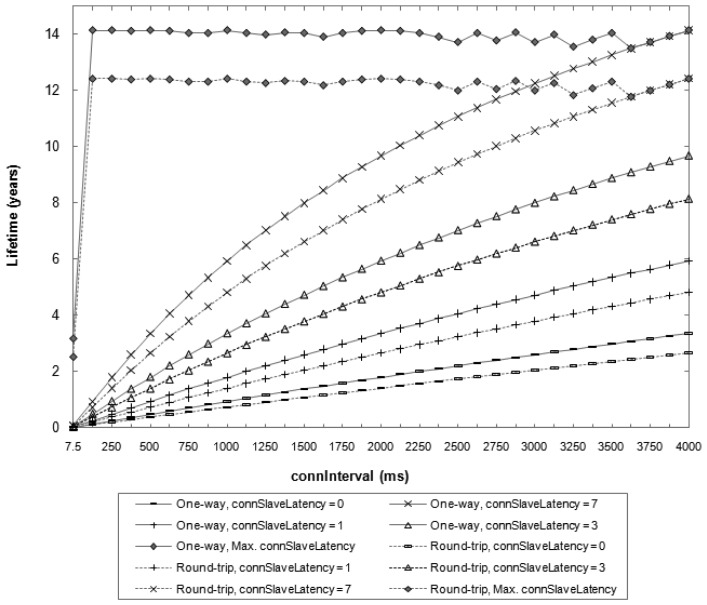
Theoretical lifetime of a slave for one-way and round-trip ATT message exchanges, and for different parameter configurations, based on CC2540 current measurements [11].

The study considers the impact of *connInterval* and *connSlaveLatency* parameters. The whole range of valid *connInterval* values (*i.e.*, from 7.5 ms to 4000 ms) is covered. For *connSlaveLatency*, values in the range between 0 and 7 are considered, since these values can be used for any permitted *connSupervisionTimeout* setting. The study is also carried out for the maximum possible *connSlaveLatency* value, which is given for the maximum *connSupervisionTimeout* value (*i.e.*, 32 s), and depends on the *connInterval* value. For this study, a BER equal to zero is assumed, which gives an upper bound on the slave lifetime under the described conditions. The results are shown in Figure 2.

Figure 2 illustrates the trade-off between slave lifetime and *connInterval* and *connSlaveLatency* parameters. The maximum slave lifetime obtained is 14.1 and 12.4 years for the one-way and round-trip methods, respectively. These values are achieved for both options for a *connInter*val of 86.25 ms and a *connSlaveLatency* of 370. With these settings, the master obtains one sensor measurement reading every 32 s. On the other hand, the most energy-intensive settings (*i.e.*, *connInterval* of 7.5 ms and *connSlaveLatency* of 0) yield a slave lifetime of 2.6 and 2.0 days for each method, respectively. In this case, the time between consecutive readings is equal to 7.5 ms. Thus, BLE offers the flexibility for accommodating a wide range of measurement reading frequencies, which trade for slave lifetime.

Note that for applications that can exploit the maximum *connSlaveLatency* values (*i.e.*, applications that do not require frequent measurements) slave lifetime does not exhibit a monotonical tendency with *connInterval*. This happens because the maximum *connSlaveLatency* is defined as an integer value and cannot be greater than 499.

**Figure 3.**
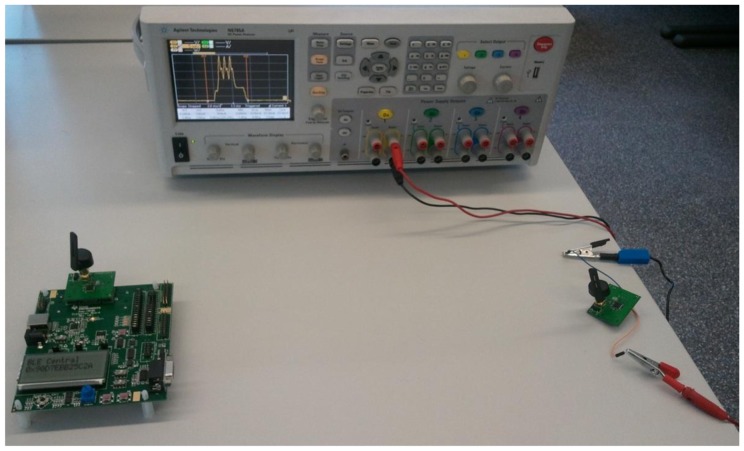
Experimental setup used for measuring the current consumption of a CC2540 slave. The devices on the left and on the right of the picture are configured as the master and the slave, respectively. The slave is connected to the power analyzer. (Note: the screen of the power analyzer shows the current consumption due to the transmission of the three advertising packets of an advertising event (see Subsection 2.3)).

**Figure 4.**
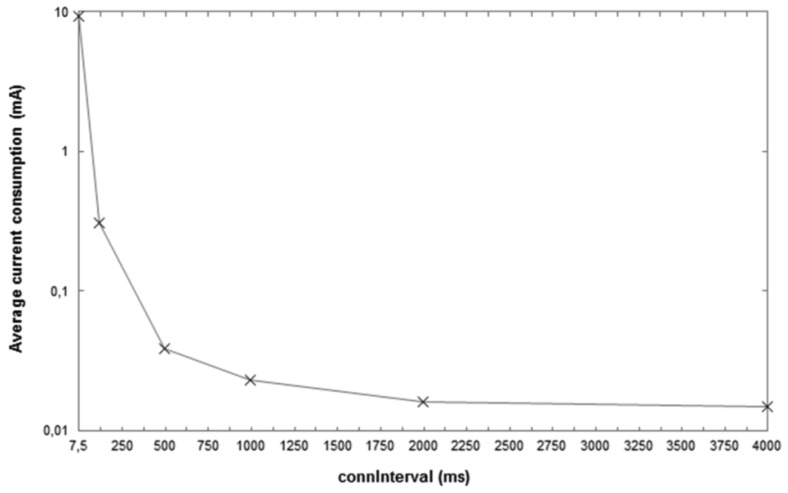
Average current consumption measured in a CC2540 slave, for the ATT one-way communication and *connSlaveLatency* = 0.

In order to complement the previous study, we also measure the average current consumption of a CC2540 node configured as a slave for the one-way ATT communication. The distance between the slave and the master is 0.5 m, and the transmit power of both devices is 0 dBm. The slave is powered by a coin cell battery that has a capacity of 220 mAh and a nominal voltage of 3 V. Measurements are carried out by using an Agilent Technologies Power Analyzer (N6705A model). Figure 3 shows a picture of the experimental setup used to perform the described current consumption measurements. Figure 4 plots the measurement results obtained, for a set of *connInterval* values within the range from 7.5 ms to 4,000 ms, and for a *connSlaveLatency* of 0.

As it can be seen, the device lifetime results shown in Figure 2 are consistent with the average current consumption results plotted in Figure 4. As *connInterval* increases, the average current consumption decreases, since the slave remains in sleep mode for a greater fraction of the connection event.

**Figure 5.**
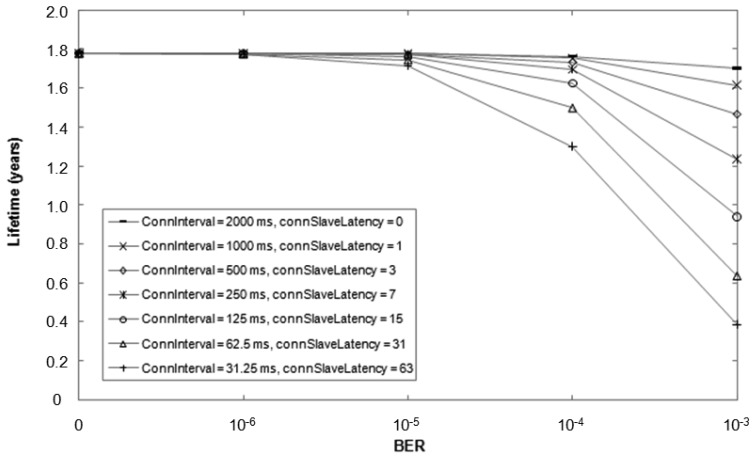
Theoretically expected slave lifetime for various *connInterval* and *connSlaveLatency* settings (which yield a notification rate of 0.5 Hz for BER = 0), for different BER values.

It is possible to perform multiple configurations of the *connInterval* and *connSlaveLatency* parameters which yield the same rate of message exchanges between master and slave under ideal conditions (*i.e.*, BER = 0). However, bit errors may significantly affect performance, depending on each particular *connInterval* and *connSlaveLatency* tuple. When a slave does not receive a packet from the master at the beginning of a connection event (e.g., due to bit errors in the access address of the packet), the slave cannot apply slave latency and must listen for a packet from the master at the beginning of every connection event, until a packet from the master is received. This mechanism allows a fast resynchronization between master and slave, so that data can be exchanged incurring low delay, at the expense of increased energy consumption. Figure 5 illustrates the theoretically expected lifetime of a slave that transmits a notification after each poll from the master for BER values up to 10^−3^ (*i.e*., the BER for which the sensitivity is defined in BLE). Note that if BER is equal to 0, the notification rate is 0.5 Hz for all the different parameter settings considered.

The choice of appropriate parameter values should be made by taking into consideration application requirements. While delivery delay may be tolerated for certain sensor measurement reading applications, a user that presses a button in a remote control is sensitive to delays greater than 500 ms [12]. Hence, if a slave is expected to react to commands sent by the master, setting *connInterval* to a low value is a safe approach. In sensor measurement transmission applications, the *connSlaveLatency* parameter can be tuned to offer the desired frequency of measurement readings and minimize energy consumption.

### 3.2. Latency

We next study by simulation the average latency of one-way ATT communications and round-trip ATT dialogues between a master and a slave, as a function of the *connInterval* parameter, and for various BER values. Examples of the one-way ATT communications considered include the following: (i) the master polls the slave and the slave replies with a notification or a command; (ii) the master sends a notification or a command, and the slave acknowledges the master's message at the Link Layer. The round-trip ATT dialogue considered is the same as that assumed in the energy consumption evaluation.

**Figure 6.**
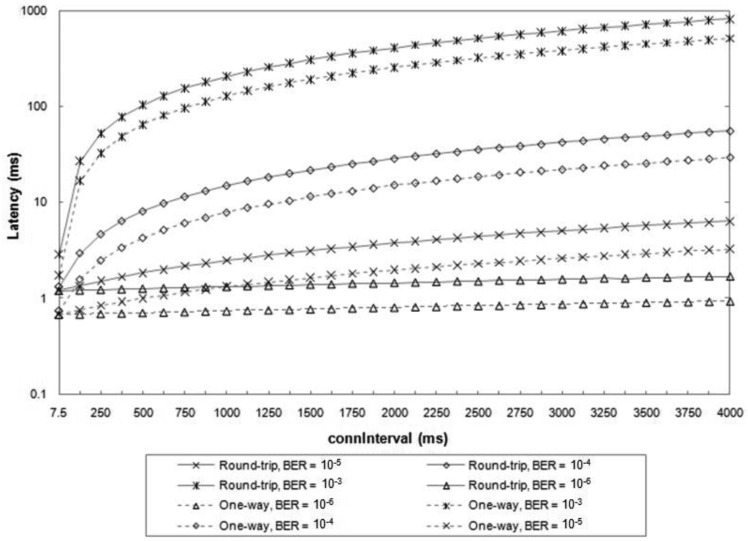
Average latency for one-way and round-trip message exchanges, for various *connInterval* and BER values.

The latency of each message exchange is measured as the difference of times between the start of the transmission of the first message and the end of the correct reception of the last message. We assume that a connection has been created between the two BLE devices before the ATT message exchange. Figure 6 illustrates the results, which are obtained as the average latency from ten million simulated message exchanges for each set of conditions. A *connSlaveLatency* equal to 0 is assumed. The results do not include the latency for the first packet of the connection. In fact, the master has flexibility in selecting the start time of the first packet transmission, which can occur between 1.25 ms and 11.25 + *connInterval* ms after the transmission of the Connection Request message.

For very low BER values (e.g., 10^−6^), the average latency of round-trip and one-way ATT message exchanges are smaller than 2 ms and 1 ms, respectively, for any *connInterval* value. However, for greater BER values, the influence of *connInterval* becomes significant, since on average more than a single connection event is required for successful transmission of each ATT message. For high BER values (e.g., 10^−3^), the average latency increases by up to three orders of magnitude.

**Figure 7.**
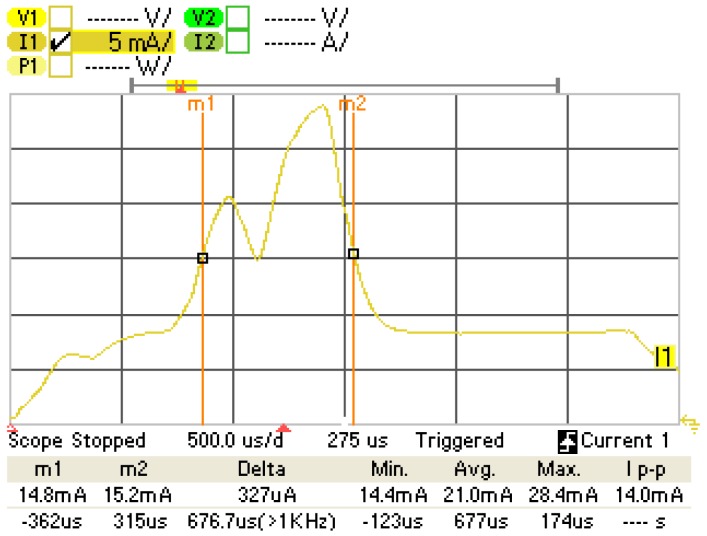
Latency measurement of a one-way ATT exchange, performed on the basis of a CC2540 slave current consumption plot. The marker m1 is placed at the start of the reception of the poll packet from the master. The marker m2 is placed at the end of the notification transmission.

On the other hand, we have measured the latency of an error-free one-way ATT exchange during the experiments presented in Subsection 3.1. The latency value obtained is 676.7 μs, which is consistent with the delay expected to transmit a maximum-sized notification, receive the corresponding acknowledgment and wait for two IFS intervals. The measurement has been performed on the basis of a CC2540 slave current consumption plot, which is illustrated in Figure 7.

### 3.3. Maximum Piconet Size

We next investigate the maximum piconet size, *i.e.*, the maximum number of slaves that a master can handle. In BLE, each connection between a master and a slave is identified by a 32-bit access address. Beyond this fact, the Bluetooth 4.0 specification does not impose further limits on the number of slaves that can be connected to a master. However, there exist practical limits on that number, depending on the type of communication between master and slave, on the *connInterval* parameter setting and the BER that can be assumed. The maximum piconet size is independent of the *connSlaveLatency* parameter, because the inactive connection events due to slave latency cannot be used for connections with other slaves.

**Figure 8.**
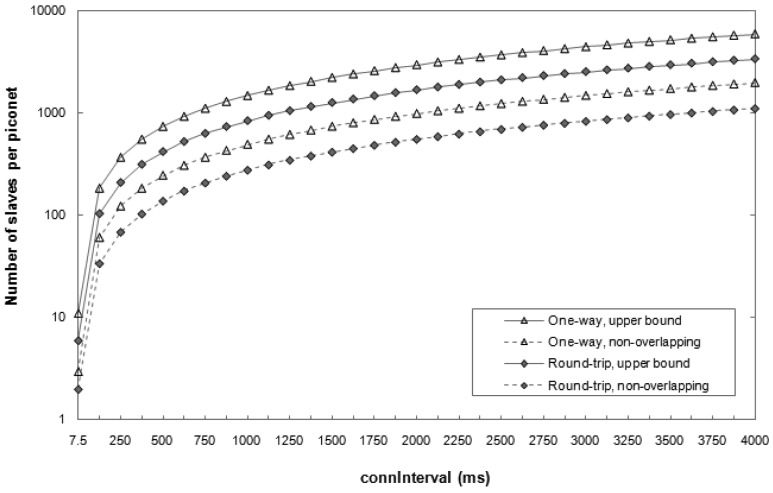
Theoretical maximum number of slaves per piconet for various types of interactions between devices and scheduling schemes.

Figure 8 depicts the theoretical maximum number of slaves that a master can handle for various configurations. This number is evaluated for the one-way and round-trip ATT interactions considered in the latency study. An upper bound on the maximum number of slaves per master is obtained by considering ideal communications (*i.e*., BER = 0). In addition, a safe scheduling scheme has been evaluated, whereby the communications between a master and two different slaves cannot overlap even when bit errors lead to retransmissions.

As shown in Figure 8, the number of slaves that a master can handle varies significantly depending on the setting of the *connInterval* parameter. The most energy-demanding configurations (*i.e*., those that use the lowest *connInterval* values) yield a limited piconet size. For example, for *connInterval* equal to 7.5 ms, the number of slaves per master is between 2 and 11, depending on the scheme considered. On the other hand, the maximum number of slaves a master can obtain data from is 5917, in the one-way ATT communication and for *connInterval* equal to 4 s.

Nevertheless, the maximum piconet size in a real scenario may be smaller than the theoretical one, due to limitations in terms of memory or antenna availability in the master. It is expected that a computer or a smartphone acting as a master will not suffer significant memory limitations. However, in some current smartphone models, the antenna is used by the classic Bluetooth and the WiFi stacks in addition to the BLE one, thus reducing the total time available for BLE and the number of possible simultaneous BLE connections.

### 3.4. Throughput

The maximum BLE application layer throughput for a connection between two devices has been obtained in prior work by simulation and mathematical analysis, as a function of *connInterval* and BER [13]. Whereas the physical layer data rate is 1 Mbps, the maximum application layer throughput is equal to 236.7 kbps. On the other hand, recall that if any of the two connected devices receives two consecutive packets with an invalid CRC check, the connection event ends. Thus, the transmission of data cannot be resumed until the beginning of the next connection event, whereby a new data channel (*i.e.*, a new frequency) is used. This behavior prevents any unnecessary waste of energy while bit errors are being found in one data channel. However, this scheme degrades the effective throughput of BLE in the presence of bit errors. In such conditions, moderate to high throughput can only be achieved for very low *connInterval* values.

Despite the theoretical or simulation-based findings, the maximum BLE throughput that can be achieved in a real scenario may be limited due to a variety of factors. These include implementation constraints such as the number of application layer messages a device can send per connection event (due to memory limitations), as well as processing delays.

We have conducted experiments in order to measure the maximum achievable throughput in a BLE link composed of two connected CC2540 devices. The distance between both devices is 0.5 m and their transmit power is 0 dBm. According to our measurements, in these conditions, the BER of the BLE link is lower than 10^−5^. In each experiment, the slave has been programmed to send the maximum number of notifications allowed by the BLE stack currently used in the CC2540 (*i.e*., four notifications per connection interval). Accordingly, we have set *connInterval* and *connSlaveLatency* to the smallest possible values (*i.e.*, 7.5 ms and 0, respectively). Each notification has the maximum size (*i.e*., 20 bytes of application layer payload). The experiment is carried out for 1000 connection events. In the described conditions, the maximum application layer throughput we have measured is 58.48 kbps. This low result can be explained by the following two facts: (i) whereas, in theory, up to eleven such notifications can be transmitted within a connection event of 7.5 ms, only four notifications are allowed per connection event, as aforementioned; and (ii) we have observed that less than four notifications are actually transmitted in most connection events during the experiment (however, the same phenomenon occurs less frequently for connection intervals greater than 7.5 ms). These observations show that high throughput has not been a primary goal in the design of the BLE implementation used in the evaluation.

## 4. Applications and Market Adoption

BLE may benefit from the widespread use of Bluetooth technology, since BLE easily integrates into classic Bluetooth circuitry, and hence it is likely that future Bluetooth devices will be dual-mode devices. According to published forecasts, BLE is expected to be used in billions of devices in the near future [4]. In view of the important role that BLE may play in the Internet of Things, the IETF 6LoWPAN WG is developing a specification in order to enable end-to-end IP communications for BLE devices [6]. For example, BLE-equipped smartphones can act as IP routers for BLE-enabled sensors and actuators. IP connectivity may dramatically increase the potential space of services and added value for BLE devices.

While BLE is emerging, other low-power wireless technologies, such as ZigBee, 6LoWPAN or Z-Wave, have already achieved significant presence in several market segments. However, they do not have high deployment expectations in devices such as smartphones. BLE, on the other hand, is expected to have a strong position in these. Table 2 shows the main characteristics of BLE and the aforementioned technologies (classic Bluetooth has been included for comparison purposes).

Healthcare, wellness and sports constitute an application domain where classic Bluetooth has already been used [14,15], and for which BLE constitutes an improvement [16]. In fact, the Continua Health Alliance, an industry coalition of healthcare and technology companies [17], announced the selection of BLE for “activity monitors and heart rate sensors to be used to monitor a user's health and fitness levels” [18].

Another opportunity for BLE is the possibility that the mobile phone may constitute a universal remote control for the domestic devices in a home. Smart energy and home security applications may further exploit BLE-enabled mobile phones. The single-hop scope of BLE may require proper transmit power tuning. In fact, most Wireless Home Automation Network (WHAN) technologies support the formation of multihop networks [2]. The dimensions of a home and signal propagation therein will determine the appropriate transmit power setting for BLE devices and whether or not BLE will be the only technology used.

On the other hand, BLE can be used for contactless applications, such as mobile payment, ticketing or access control. However, Near Field Communication (NFC) was primarily designed for this use case space [9, 19], and since then, the panoply of device models that support NFC has been growing at a moderate but steady rate. NFC defines a subhundred-millisecond setup, two-way wireless technology based on the principle of magnetic inductive coupling, which allows for very short range (typically, below 20 cm). Therefore, NFC offers very limited device physical location flexibility in comparison with BLE, while it also offers significantly reduced security threat scope. Since transactions in the aforementioned application space are critical from a security viewpoint, the use of BLE would require the Passkey Entry or an adequate Out of Band pairing method. Given the properties of NFC, it constitutes a powerful candidate as Out of Band technology for BLE pairing, which additionally avoids the need to enter a key by the user. This combination of BLE and NFC provides advantages of both technologies. Note that NFC does not impact on BLE use cases whereby communication range is greater than a few tens of centimeters.

BLE is also adequate for industrial environments, which are challenging due to multipath fading and radio interference from machinery [20]. Inherited from classic Bluetooth, BLE accounts with adaptive frequency hopping, which offers a robust solution to the aforementioned problems. Frequency hopping is also used in industrial standards such as WirelessHART and ISA 100.11a, and is a component of the recently published IEEE 802.15.4e specification [21].

## 5. Conclusions

This paper describes the BLE protocol stack, provides a performance evaluation of this technology and explores its potential applications. In BLE, there exists a trade-off between energy consumption, latency, piconet size, and throughput that mainly depends on the *connInterval* and *connSlaveLatency* parameters. Evaluation results show how these parameters can be tuned wisely in order to meet application requirements. On the other hand, the paper points out several implementation constraints that may reduce BLE performance in a real scenario, in comparison with the theoretically expected one. BLE emerges as a strong low-power wireless technology for single-hop communication use cases which may contribute to connecting a dramatically large amount of new devices to the Internet of Things.

## Figures and Tables

**Table 1.** Security services and features for the security modes and levels defined in BLE.

		**Pairing**	**Encryption**	**Data Integrity**	**Layer**

**LE Security Mode 1**	**Level 1**	No	No	No	Link Layer
	**Level 2**	Unauthenticated	Yes	Yes	
	**Level 3**	Authenticated	Yes	Yes	

**LE Security Mode 2**	**Level 1**	Unauthenticated	No	Yes	ATT layer
	**Level 2**	Authenticated	No	Yes	

**Table 2.** Main characteristics of ZigBee, 6LoWPAN, Z-Wave, BLE and classic Bluetooth. The data of ZigBee, 6LoWPAN and Z-Wave has been obtained or adapted from the literature [2].

		**ZigBee**	**6LoWPAN (Over 802.15.4)**	**Z-Wave**	**Bluetooth Low Energy**	**Classic Bluetooth**
**Physical layer**	**RF band (MHz)**	868/915/2400		868/908 (all chips) 2400 (400 serie chip)	2400	2400
	**Bit rate (kbps)**	20/40/250		9.6/40 (from 200 series chip) 200 (only 400 series chip)	1000	≤721(v1.2), 3000 (v2+EDR), ≤24,000 (v3+HS)
	**Modulation**	BPSK/BPSK/O-QPSK		BFSK	GFSK	GFSK (v1.2), GFSK/π/4-DQPSK/8DPSK (v2+EDR), 802.11 (v3+HS)
	**Spreading technique**	DSSS		No	FHSS (2 MHz channel width)	FHSS(1 MHz channel width)
	**Receiver sensitivity (dBm)**	−85 or better(2.4 GHz band)−92 or better(868/915 MHz bands)		−101 (at 40 kbps)	≤−70(required)	
					−87 to −93 (typical)	−90(typical)
	**Transmit power (dBm)**	−32 to 0		−20 to 0	−20 to 10	20/4/0(Class 1/2/3)
**Link layer**	**MAC mecha-nism**	TDMA+CSMA/CA (beacon mode) and CSMA/CA (beaconless mode)		CSMA/CA	TDMA	TDMA
	**Message size (bytes)**	127 (maximum)		64 (max. MAC payload in 200 series chip)	8 to 47	358 (maximum)
	**Error control**	16-bit CRC. ACKs (optional)		8-bit checksum. ACKs (optional)	24-bit CRC. ACKs	8-bit CRC (header); 16-bit CRC and 2/3 FEC (payload). ACKs
	**Latency (ms)**	<5 (beaconless mode, at 250 kbps)		<39 (at 40 kbps)	<3	<100
**Identifiers**		16- and 64-bit MAC addresses. 16-bit NWK identifiers	16- and 64-bit MAC addresses. 128-bit IPv6 addresses	32-bit (home ID), 8-bit (node ID)	48-bit public device Bluetooth address or random address	48-bit public device Bluetooth address
**Device types or roles**		Coordinator, Router and End device	Edge Router, Mesh Node (mesh under), Router (route over), Host	Controller and slave	Master and slave	Master and slave
**Network layer**	**Multi-hop solution**	Mesh routing, tree routing, and source routing	RPL (other protocols are not excluded)	Source routing	Not currently supported	Scatternet (routing protocol out of the scope of the Bluetooth specifications)
	**Hop limit**	30/10/5 (mesh routing/tree routing/source routing)	255	4	1	Outside scope of Bluetooth specifications
**Security**		Integrity, confidentiality, access control (IEEE 802.15.4 security, using 128-bit AES)		128-bit AES encryption (400 series chip)	Security Modes/Levels. Pairing. Key Gener./Distribution. Confidentiality, Authentication, and Integrity	Pairing and Link Key Generation. Authentication. Confidentiality. Trust Levels, Service Levels, and Authorization. *E_x_* algorithms
		Key management	Key management currently out of scope			
**Implementation size**		45–128 kB (ROM), 2.7–12 kB (RAM)	24 kB (ROM), 3.6 kB (RAM)	32–64 kB (Flash), 2–16 kB (SRAM)	∼40 kB (ROM), ∼2.5 kB (RAM)	∼100 kB (ROM), ∼30 kB (RAM)

## References

[1] (2010). Specification of the Bluetooth System, Covered Core Package, Version: 4.0.

[2] Gomez C., Paradells J. (2010). Wireless home automation networks: A survey of architectures and technologies. IEEE Commun. Mag..

[3] Ludovici A., Calveras A., Casademont J. (2011). Forwarding techniques for IP fragmented packets in a real 6LoWPAN network. Sensors.

[4] West A. Smartphone, the key for Bluetooth low energy technology. http://www.bluetooth.com/Pages/Smartphones.aspx.

[5] Hui J.W., Culler D.E. (2008). Extending IP to low-power, wireless personal area networks. IEEE Internet Comput..

[6] Nieminen J., Patil B., Savolainen T., Isomaki M., Shelby Z., Gomez C. (2012). Transmission of IPv6 packets over Bluetooth low energy draft-ietf-6lowpan-btle-08.

[7] Haartsen J.C. (2000). The Bluetooth radio system. IEEE Pers. Commun..

[8] Zheng J., Lee M.J., Anshel M. (2006). Towards secure low rate wireless personal area networks. IEEE Trans. Mob. Comput..

[9] Echevarría J.J., Ruiz-de-Garibay J., Legarda J., Álvarez M., Ayerbe A., Vázaquez J.I. (2012). WebTag: Web browsing into sensor tags over NFC. Sensors.

[10] Callegati F., Cerroni W., Ramili M. (2009). Man-in-the-Middle attack to the HTTPS protocol. IEEE Secur. Priv..

[11] Kamath S. (2010). Measuring Bluetooth Low Energy Power Consumption.

[12] Ko J., Terzis A., Dawson-Haggerty S., Culler D.E., Hui J.W., Levis P. (2011). Connecting low-power and lossy networks to the internet. IEEE Commun. Mag..

[13] Gomez C., Demirkol I., Paradells J. (2011). Modeling the maximum throughput of Bluetooth low energy in an error-prone link. IEEE Commun. Lett..

[14] Chang K.-M., Liu S.-H., Wu X.-H. (2012). A Wireless sEMG recording system and its application to muscle fatigue detection. Sensors.

[15] Nakamura M., Nakamura J., Lopez G., Shuzo M., Yamada I. (2011). *C*ollaborative processing of wearable and ambient sensor system for blood pressure monitoring. Sensors.

[16] Patel M., Wang J. (2010). Applications, challenges, and prospective in emerging body area networking technologies. IEEE Commun. Mag..

[17] Carroll R., Cnossen R., Schnell M., Simons D. (2007). Continua: An interoperable personal healthcare ecosystem. IEEE Pervasive Comput..

[18] Helge Omre A. (2010). Bluetooth low energy: Wireless connectivity for medical monitoring. J. Diabetes Sci. Technol..

[19] Ortiz S. (2006). Is Near-Field Communication Close to Success?. IEEE Comput..

[20] Tinka A., Watteyne T., Pister K., Bayen A.M. (2010). A decentralized scheduling algorithm for time synchronized channel hopping. Ad Hoc Netw..

[21] Silva I., Guedes L.A., Portugal P., Vasques F. (2012). Reliability and availability evaluation of wireless sensor networks for industrial applications. Sensors.

